# Eating behaviors and liver health in metabolic dysfunction-associated fatty liver disease: the serial mediating roles of Mediterranean diet adherence and adiposity

**DOI:** 10.3389/fnut.2026.1740363

**Published:** 2026-02-10

**Authors:** Büşra Başar Gökcen, İrem Kurtuluş, Nermin Kabak, Ferenc Budan, Duygu Ağagündüz, Dávid Szép

**Affiliations:** 1 Department of Nutrition and Dietetics, Fethiye Faculty of Health Sciences, Muğla Sıtkı Koçman University, Muğla, Türkiye; 2 Medical School, Institute of Physiology, University of Pécs, Pécs, Hungary; 3 Department of Nutrition and Dietetics, Faculty of Health Sciences, Gazi University, Ankara, Türkiye

**Keywords:** adiposity, eating behavior, liver disease, Mediterranean diet, obesity

## Abstract

**Background:**

This study aimed to investigate the mediating roles of healthy diet adherence and adiposity in the relationship between eating behaviors (uncontrolled or mindful eating) and liver health according to metabolic dysfunction-associated fatty liver disease (MALFD) status.

**Methods:**

Adults with and without MAFLD (150 and 90, respectively) were included. Eating behaviors were assessed using the Mindful Eating Questionnaire (MEQ) and Three-Factor Eating Questionnaire (TFEQ), and Mediterranean diet adherence was measured with the Mediterranean Diet Adherence Screener (MEDAS). Adiposity indices included Body Mass Index (BMI) and Visceral Adiposity Index (VAI). Liver health markers were alanine aminotransferase (ALT) and aspartate aminotransferase (AST). Statistical analyses comprised group comparisons, Spearman correlations, and serial mediation models tested using Hayes’ PROCESS macro.

**Results:**

Individuals with MAFLD had higher BMI (median 32 vs. 25 kg/m^2^), VAI (4.1 vs. 1.1), and ALT levels (83 vs. 20 U/L, all *p* < 0.001). In unadjusted comparisons, the MAFLD group showed lower MEQ and MEDAS scores, together with higher emotional eating and uncontrolled eating scores on the TFEQ. In multivariable logistic regression analyses, BMI was independently associated with MAFLD status (OR = 1.43, 95% CI: 1.30–1.57), while eating behavior scores were not significant after BMI adjustment. Serial mediation analyses using PROCESS Model 6 showed that the association between uncontrolled eating scores and ALT levels was indirectly transmitted through BMI, with the serial indirect pathway involving MEDAS and BMI reaching statistical significance in the MAFLD group (indirect effect = 0.106; 95% bootstrap CI: 0.019–0.251). Moderated mediation analyses using PROCESS Model 92 further indicated a significant index of moderated mediation for this pathway (IMM = 0.090; 95% bootstrap CI: 0.003–0.233), whereas mediation models based on mindful eating scores did not yield significant moderated mediation effects.

**Conclusion:**

The results support the presence of indirect associations linking eating behavior scores to liver enzyme levels via Mediterranean diet adherence and adiposity, particularly in the context of MAFLD. These pathways point to potentially modifiable behavioral and dietary targets, while underscoring the need for confirmation through prospective and interventional studies.

## 1 Introduction

MAFLD, previously referred to as non-alcoholic fatty liver disease (NAFLD), is one of the most common chronic liver diseases worldwide, affecting approximately 30% of the adult population. With its rapidly increasing prevalence, MAFLD has become a major public health concern and a leading cause of liver-related morbidity and mortality. It is closely associated with lifestyle-related metabolic disorders, including obesity, type 2 diabetes mellitus, and cardiovascular diseases (1–4). This disease encompasses a broad spectrum of liver pathologies, ranging from simple hepatic steatosis, defined as fat accumulation in more than 5% of hepatocytes in the absence of significant alcohol consumption, that is less than 20 grams per day for women and 30 grams per day for men, and other chronic liver diseases, to non-alcoholic steatohepatitis (NASH) and advanced fibrosis (5, 6).

The pathogenesis of MAFLD is no longer considered a process limited to hepatic fat accumulation alone; instead, it is increasingly understood through the lens of the “multiple hit” hypothesis. According to this model, MAFLD is a complex and systemic disease shaped by the interplay of multiple factors, including insulin resistance, ectopic fat accumulation, oxidative stress, inflammatory cytokine activation, gut microbiota alterations, and genetic predisposition. Consequently, its treatment requires a comprehensive and multidisciplinary approach (7).

There is currently no pharmacological treatment with proven efficacy for the management of MAFLD. Therefore, leading clinical guidelines, including those issued by the European Society for Clinical Nutrition and Metabolism (ESPEN), the American Association for the Study of Liver Diseases (AASLD), the European Association for the Study of the Liver (EASL), and the American Association of Clinical Endocrinology (AACE), recommend lifestyle modifications as the cornerstone of MAFLD treatment. In particular, a hypocaloric diet creating a daily energy deficit of 500 to 1,000 kcal combined with moderate intensity physical activity is considered the most effective approach for achieving sustained weight loss. Weight reduction of at least 5% and preferably 10% has been associated with significant improvements in hepatic steatosis, liver histopathology, and overall cardiometabolic risk. In addition, strong recommendations support the adoption of the Mediterranean diet due to its antioxidant and anti-inflammatory properties, as well as its beneficial effects on insulin sensitivity and hepatic fat accumulation (8–13).

The Mediterranean diet is characterized by limited intake of saturated fats and animal protein, a high content of antioxidants, dietary fiber, and monounsaturated fatty acids (MUFA), and a balanced ratio of omega 3 to omega 6 fatty acids. This dietary pattern has been well documented for its effectiveness in preventing cardiovascular disease, metabolic syndrome, and type 2 diabetes. Moreover, it has been shown to exert protective and regulatory effects in the context of MAFLD (14–16). However, several factors, including limited nutritional knowledge, low intrinsic motivation, emotional stress, socio-cultural influences, and incompatibility with habitual eating patterns, negatively affect adherence to the Mediterranean diet among individuals with MAFLD. Conversely, facilitating factors that enhance dietary adherence include mindful eating, an internal locus of control, and the ability to establish a balanced relationship with food (17).

Mindfulness is defined as a present-centered awareness of bodily sensations, cognitive and emotional processes, and experiences in the external environment, approached with a nonjudgmental, accepting, and equanimous attitude. The cultivation of this capacity is associated with self-regulatory processes that integrate attentional control and emotional regulation, thereby supporting behavior change. Indeed, mindful eating emphasizes intentionally paying attention to food and the eating experience moment by moment and without judgment, with a focus on sensory awareness and full presence rather than on calorie content or specific macronutrients. Mindfulness-based eating approaches further emphasize internal bodily cues over external eating triggers, thereby supporting appetite regulation within a framework that acknowledges the complex interplay between physiological and psychological processes (18–20). In contrast, dysfunctional eating patterns such as emotional and uncontrolled eating may hinder the adoption of sustainable dietary behaviors. Uncontrolled eating, characterized by a loss of control and recurrent overeating despite efforts to restrain intake, and emotional eating, defined as eating in response to emotional states rather than physiological hunger, are commonly triggered by emotional or external cues, including stimuli such as observing others eat. These eating patterns, which are triggered by emotional or external cues rather than physiological hunger, including stimuli such as observing others eat, are commonly associated with a preference for energy-dense foods high in fat and sugar and with unhealthy food choices (21–23).

Taken together, these contrasting eating patterns highlight mindful eating as a key behavioral mechanism supporting nutritional self-regulation, while emotional and uncontrolled eating emerge as important targets for intervention in efforts to promote sustainable dietary change. Building on this background, the present study aimed to examine how eating behaviors, particularly mindful eating and uncontrolled eating, are linked to liver health by testing whether Mediterranean diet adherence and BMI act as sequential mediators, and whether these pathways differ according to MAFLD status.

## 2 Materials and methods

### 2.1 Study design and population

This study was designed as an observational, analytical, and cross-sectional research. It was conducted at the Internal Medicine Outpatient Clinic of Fethiye State Hospital, affiliated with the Muğla Provincial Health Directorate, between October 2023 and January 2024. Participants were recruited through referrals from specialist physicians working in the outpatient clinic. The research team contacted referred individuals in the outpatient clinic waiting area and conducted the information and enrollment procedures without interfering with routine clinical care. Participants were informed about the purpose and scope of the study, and written informed consent was obtained prior to participation. As the research team did not include personnel qualified to perform diagnostic assessments, no diagnostic procedures were conducted within the scope of the study. Accordingly, MAFLD status was based on participants’ report of a physician-confirmed diagnosis established by specialist physicians during routine outpatient clinical evaluation, including assessment of metabolic risk factors, laboratory findings, and, where applicable, imaging modalities such as abdominal ultrasonography, in accordance with standard clinical practice. Data were collected by a trained researcher through face-to-face interviews conducted under standardized conditions.

Individuals who applied to the Internal Medicine Outpatient Clinic, were aged 18 years or older, voluntarily agreed to participate, and were classified according to physician-confirmed MAFLD status were included in the study. Participants with MAFLD were assigned to the MAFLD group, whereas those without a diagnosis were classified as the non-MAFLD group. The non-MAFLD group consisted of individuals who attended the Internal Medicine Outpatient Clinic for routine examinations, general health evaluations, or non-metabolic reasons, and were confirmed not to meet MAFLD diagnostic criteria based on routine clinical evaluation, laboratory findings, and medical history. Individuals classified as non-MAFLD had no prior physician-diagnosed metabolic disease, including type 2 diabetes mellitus, hypertension, dyslipidemia, cardiovascular disease, or metabolic syndrome, and did not meet the diagnostic criteria for MAFLD based on routine clinical evaluation. Individuals under the age of 18, pregnant or breastfeeding women, those with a history of alcohol consumption (more than 10 g/day for women and 20 g/day for men), those with complications related to MAFLD, and those unable to complete the questionnaire due to physical, cognitive, or psychological limitations were excluded from the study. A total of 240 participants were included in the study, comprising 150 individuals with MAFLD and 90 individuals without MAFLD.

The study was approved by the Ethics Committee of Muğla Sıtkı Koçman University Faculty of Medicine and Health Sciences on February 16, 2023, with protocol-decision number 220169–26. In addition, institutional permission was obtained from the Muğla Provincial Health Directorate on June 16, 2023.

### 2.2 Data collection procedures and tools

Data were collected using a structured questionnaire composed of four main sections. The first section included sociodemographic and anthropometric data; the second section covered eating habits; the third comprised validated self-report scales; and the fourth included clinical and biochemical indicators.

#### 2.2.1 Anthropometric measurements

Anthropometric assessments were conducted using standardized procedures and calibrated tools, ensuring measurement accuracy and reliability. All measurements were performed by trained personnel with participants standing in an upright position, barefoot, and in light clothing. Body weight was measured to the nearest 0.1 kg using a calibrated digital scale, while height was measured to the nearest 0.1 cm using a stadiometer. BMI was calculated using the standard formula: weight (kg) / height^2^ (m^2^). Waist Circumference (WC) was measured with a non-elastic, flexible measuring tape placed horizontally midway between the lowest rib and the iliac crest, at the end of a normal expiration. The measurement was recorded to the nearest 0.1 cm. Hip Circumference (HC) was measured at the point of the maximum circumference over the buttocks, using the same non-stretchable tape. Participants were asked to stand with feet together and arms at their sides during measurement. Waist-to-Hip Ratio (WHR) was calculated by dividing waist circumference (cm) by hip circumference (cm) (24).

#### 2.2.2 Mindful eating questionnaire

The MEQ was originally developed by Framson et al. (2009) as a 28-item instrument designed to assess mindful eating behaviors (25). Subsequently, five items from the original scale were retained, while the remaining items were adapted and expanded upon, resulting in a revised version consisting of 30 items. The adapted version is scored on a 5-point Likert scale (1 = never, 5 = always) and includes seven subscales: disinhibition, emotional eating, eating control, attention/focus, eating discipline, awareness, and interference. Higher scores in each subscale indicate a greater presence of problems related to that specific domain. As the score increases, the severity of the issue in that subscale also rises (26–28). The Turkish validity and reliability of the adapted version were confirmed by Köse et al. in 2016 (26).

#### 2.2.3 Three-factor eating questionnaire

The TFEQ was originally developed by Stunkard and Messick in 1985 to assess the behavioral and cognitive components of eating. The original version of the scale consisted of 51 items divided into two sections and evaluated eating behavior through three subscales (29). The scale was later revised and shortened to 18 items by Karlsson et al. in 2000, while maintaining the original three-factor structure (30). In subsequent studies, the revised version was further expanded to include 21 items (31). The Turkish validity and reliability of the original 51-item form were established by Baş et al. in 2008 (32). The revised 21-item version, which retains the three original factors—emotional eating, cognitive restraint, and uncontrolled eating—(33) was adapted into Turkish culture and validated by Karakuş et al. in 2016 (34). In the present study, the 21-item version of the TFEQ was used, as this version has demonstrated established validity and reliability in the Turkish population and retains the original three-factor structure of the scale.

#### 2.2.4 Mediterranean diet adherence screener

Adherence to the Mediterranean dietary pattern was assessed using the 14-item MEDAS, originally developed by Martínez-González et al. within the PREDIMED trial (35). The Turkish validation and reliability study was conducted by Pehlivanoğlu et al. (36). The total score ranges between 0 and 14. Higher scores indicate greater adherence to the Mediterranean diet. A MEDAS score below 7 was classified as “unacceptable diet adherence”, whereas a score of 7 or above was considered “acceptable diet adherence” (36).

#### 2.2.5 Biochemical analyses

Biochemical analyses were performed using venous blood samples collected from participants as part of their routine health assessments. Blood samples were drawn in the morning following an overnight fasting period of at least 8–12 h. On the day of collection, blood samples were centrifuged to separate serum and subsequently analyzed in the clinical laboratory.

Following centrifugation, biochemical parameters were analyzed using spectrophotometric methods on a Roche Cobas C501 automated analyzer (Roche Diagnostics, COBAS C501, Indianapolis, IN, United States). Within this scope, liver function tests included the measurement of alanine aminotransferase (ALT) and aspartate aminotransferase (AST) levels. In addition, serum total cholesterol, low-density lipoprotein (LDL) cholesterol, high-density lipoprotein (HDL) cholesterol, and triglyceride (TG) levels were assessed.

The Visceral Adiposity Index (VAI) was calculated separately for men and women based on WC, BMI, triglyceride (TG), and HDL cholesterol levels, using the following formulas (37).

$\begin{array}{l} \text{For women}:(\mathrm{WC}/[36.58+(1.89\times \mathrm{BMI})])\times \\ (\mathrm{TG}/0.81)\times (1.52/\mathrm{HDL}). \end{array}$

$\begin{array}{l} \text{For}\;\mathrm{men}:(\mathrm{WC}/[39.68+(1.88\times \mathrm{BMI})])\times \\ (\mathrm{TG}/1.03)\times (1.31/\mathrm{HDL}). \end{array}$

#### 2.2.6 Statistical analysis

All analyses were conducted using IBM SPSS Statistics version 30.0 and the PROCESS macro version 4.2 by Andrew F. Hayes (38). A two-tailed *p* < 0.05 was considered statistically significant. Continuous variables were summarized as median (IQR), and categorical variables as percentages (%). Normality was evaluated using the Shapiro–Wilk test, histograms, Q–Q plots, and skewness–kurtosis coefficients. Between-group comparisons were performed using the Mann–Whitney U test for continuous variables. Potential confounding by age, sex, and BMI was adjusted using analysis of covariance (ANCOVA). Associations between study variables were examined using Spearman’s correlation coefficients (r) with corresponding *p* values.

Multivariable logistic regression analysis was performed to identify factors independently associated with MAFLD status (dependent variable; coded as 1 = MAFLD and 0 = non-MAFLD). In Model 1, eating behavior–related variables, including mindful eating (MEQ global score), uncontrolled eating (TFEQ UE subscale), and Mediterranean diet adherence (MEDAS global score), were entered simultaneously. In Model 2, body mass index (BMI, kg/m^2^) was additionally included to evaluate its contribution beyond eating behavior variables. Results are presented as odds ratios (ORs) with 95% confidence intervals (95% CI). Model fit was assessed using Cox & Snell and Nagelkerke pseudo R^2^ statistics.

To investigate the sequential pathways linking eating behaviors to liver-related outcomes, serial mediation analyses were conducted using the PROCESS macro (Model 6). Separate models were specified for mindful eating and uncontrolled eating as independent variables (X), with Mediterranean diet adherence (MEDAS) entered as the first mediator (M1), body mass index (BMI) as the second mediator (M2), and serum alanine aminotransferase (ALT) as the dependent variable (Y). Direct, indirect, and total effects were estimated simultaneously. The significance of indirect effects was evaluated using bias-corrected percentile bootstrap confidence intervals based on 5,000 resamples, with effects considered statistically significant when the 95% confidence interval did not include zero. To assess whether direct and indirect effects differed according to MAFLD status, moderated serial mediation analyses were performed using PROCESS Model 92. MAFLD status was specified as the moderator (W), and interaction effects were evaluated using the index of moderated mediation (IMM) with 95% bootstrap confidence intervals.

ALT was selected as the primary outcome variable due to its higher liver specificity and sensitivity to hepatocellular injury in MAFLD, while BMI was specified as a key metabolic mediator reflecting the cumulative impact of eating behaviors and dietary patterns on body composition. Age and sex were included as covariates in all regression models. Results are reported as unstandardized regression coefficients (B), corresponding *p* values, and 95% confidence intervals, as appropriate.

An *a priori* power analysis was conducted using GPower version 3.1 based on the primary group comparison between MAFLD and non- MAFLD participants. Assuming a medium effect size (Cohen’s d = 0.50), an alpha level of 0.05, and a desired statistical power of 0.95, the required total sample size was calculated as 210 participants. The final sample size of the study exceeded this requirement.

## 3 Results

A total of 240 adult individuals participated in the study. Of these, 150 (62.5%) were diagnosed with MAFLD and comprised the case group, while 90 (37.5%) had no metabolic disease and comprised the control group. In the case group, 68.0% of the participants were female, whereas this proportion was 78.9% in the control group (*p* = 0.046). In terms of marital status, 83.3% of the case group were married compared to 42.2% in the control group (*p* < 0.001). Regarding educational status, 38.0% of the case group had a high level of education, while this rate was 76.7% in the control group (*p* < 0.001). The proportion of non-smokers was 82.7% in the case group and 81.1% in the control group, with no statistically significant difference (*p* = 0.445). Similarly, the proportion of non-alcohol users was 85.3% in the case group and 83.3% in the control group, and this difference was not statistically significant (*p* = 0.405).

As shown in Table 1, individuals with MAFLD differed markedly from those without MAFLD with respect to anthropometric measures and biochemical parameters. The MAFLD group exhibited higher values for age, BMI, waist, hip and neck circumferences, liver enzymes (ALT and AST), lipid profile parameters, and VAI compared with the non-MAFLD group, with all unadjusted and adjusted comparisons remaining statistically significant (*p* < 0.001).

**Table 1:** Anthropometric and biochemical profile according to MAFLD status.

Variable	MAFLD ^(n: 150)^	Non-MAFLD ^(n: 90)^	Z	F
	Median (IQR)	Median (IQR)		
Age (years)	55 (45–63)	32.5 (23–50)	−7.322	--
Body mass index (kg/m^2^)	32 (30–36)	25 (22–29)	−9.802	--
Waist circumference (cm)	115 (108–126)	82 (70–94)	−10.823	41.919
Hip circumference (cm)	120 (110–128)	95 (90–108)	−9.596	23.319
Neck circumference (cm)	43 (40–48)	32 (30–37)	−9.475	28.430
ALT (U/L)	83 (68–110)	20 (15–28)	−12.289	184.908
AST (U/L)	83 (67–104)	20 (16–26)	−12.202	171.758
Total cholesterol (mg/dL)	255 (230–293)	178.5 (160–201)	−9.707	51.266
LDL cholesterol (mg/dL)	142.5 (133–160)	98 (88–115)	−8.999	50.360
HDL cholesterol (mg/dL)	40 (35–48)	53 (47–59)	−7.871	38.152
Triglycerides (mg/dL)	177 (160–191)	110.5 (85–128)	−9.873	59.725
Visceral adiposity index	4.1 (3.2–5.3)	1.1 (0.9–1.7)	−11.843	148.946

Data are presented as median (IQR). Unadjusted group comparisons were performed using the Mann–Whitney U test (Z values). Comparisons adjusted for age, sex, and BMI were conducted using rank-based ANCOVA or generalized linear models, as appropriate (F values). All unadjusted and adjusted comparisons were statistically significant (*p* < 0.001). ALT, alanine aminotransferase; AST, aspartate aminotransferase; LDL, low-density lipoprotein; HDL, high-density lipoprotein.

As shown in Table 2, several dietary behavior indicators differed between individuals with MAFLD and those without MAFLD. In unadjusted analyses, the MAFLD group had lower MEQ global scores (*p* < 0.001) and higher emotional eating (*p* = 0.028) and uncontrolled eating (*p* = 0.009) compared with the non-MAFLD group. Mediterranean diet adherence was also lower in the MAFLD group (*p* = 0.024), whereas cognitive restraint did not differ between groups (*p* = 0.438). After adjustment for age, sex, and BMI, none of the dietary behavior indicators differed significantly between groups (all *p* > 0.05).

**Table 2:** Dietary behavior indicators according to MAFLD status.

Variable	MAFLD	non-MAFLD	Z	p^1^	F	p^2^
	Median (IQR)	Median (IQR)				
MEQ, GS	90.5 (81–101)	97 (88–109)	−3.674	**<0.001**	1.779	0.184
TFEQ, EE	15 (11–17)	13 (10–17)	−2.194	**0.028**	0.000	0.986
TFEQ, CR	15 (12–18)	15.5 (13–18)	−0.775	0.438	1.052	0.306
TFEQ, UE	23 (18–26)	20 (17–24)	−2.628	**0.009**	0.366	0.546
MEDAS, GS	7 (5–8)	7 (6–8)	−2.256	**0.024**	0.142	0.707

Data are presented as median (IQR). Unadjusted group comparisons were performed using the Mann–Whitney U test and are reported as Z values and p^1^. Comparisons adjusted for age, sex, and BMI were conducted using rank-based ANCOVA or generalized linear models, as appropriate, and are reported as F values and p^2^. MEQ, Mindful Eating Questionnaire; GS, global score; MEDAS, Mediterranean Diet Adherence Screener; TFEQ, Three-Factor Eating Questionnaire; EE, emotional eating; CR, cognitive restraint; UE, uncontrolled eating. Bold values indicate statistically significant results (*p* < 0.05).

As shown in Table 3, in Model 1, the MEQ score was significantly associated with MAFLD status (OR = 0.962, 95% CI: 0.935–0.990, *p* = 0.008). TFEQ uncontrolled eating and MEDAS scores were not significantly associated with MAFLD in this model. In Model 2A, after inclusion of BMI, BMI was significantly associated with MAFLD (OR = 1.432, 95% CI: 1.303–1.573, *p* < 0.001). In this model, MEQ, TFEQ uncontrolled eating, and MEDAS scores were not statistically significant. In Model 2B, when VAI was included instead of BMI, VAI was significantly associated with MAFLD (OR = 7.454, 95% CI: 4.522–12.286, *p* < 0.001). In this model, none of the eating behavior scores showed a statistically significant association with MAFLD status.

**Table 3:** Multivariable logistic regression analysis of factors associated with MAFLD.

Variable	Model 1			Model 2A			Model 2B		
	OR	95% CI	*p*	OR	95% CI	*p*	OR	95% CI	*p*
MEQ, GS	0.962	0.935–0.990	**0.008**	0.983	0.948–1.019	0.355	0.970	0.924–1.019	0.225
TFEQ, UE	0.982	0.922–1.046	0.567	0.964	0.890–1.044	0.368	0.955	0.861–1.060	0.389
MEDAS, GS	0.947	0.810–1.109	0.501	1.016	0.836–1.233	0.877	0.945	0.715–1.248	0.689
BMI (kg/m^2^)	-----	-----	-----	1.432	1.303–1.573	**<0.001**	-----	-----	-----
VAI	-----	-----	-----	-----	-----	-----	7.454	4.522–12.286	**<0.001**
**Model Fit (R** ^**2**^ **)**	**Cox & Snell: 0.058, Nagelkerke: 0.079**			**Cox & Snell: 0.395, Nagelkerke: 0.538**			**Cox & Snell: 0.565, Nagelkerke: 0.769**		

Data are presented as odds ratios (ORs) with 95% confidence intervals (95% CI). Model 1 included eating behavior variables only (MEQ score, TFEQ uncontrolled eating score, and MEDAS score). Model 2A additionally included BMI as an indicator of general adiposity. Model 2B replaced BMI with the VAI to assess the contribution of visceral adiposity. BMI and VAI were not entered simultaneously into the same model because they represent overlapping adiposity constructs and exhibit a high degree of collinearity. MAFLD status was coded as 1 = MAFLD and 0 = non-MAFLD. Pseudo R^2^ values (Cox & Snell and Nagelkerke) are reported to indicate model fit. MEQ, Mindful Eating Questionnaire; MEDAS, Mediterranean Diet Adherence Screener; TFEQ, Three-Factor Eating Questionnaire; UE, uncontrolled eating; GS, global score. Bold values indicate statistically significant results (*p* < 0.05).

As shown in Table 4, several significant correlations were observed between behavioral eating pattern scores and metabolic-liver parameters. MEQ total score was negatively correlated with emotional eating score (*r* = −0.647, *p* < 0.01) and uncontrolled eating score (*r* = −0.707, *p* < 0.01) and was inversely associated with the VAI and liver enzyme levels. The TFEQ uncontrolled eating score was positively correlated with the emotional eating score (*r* = 0.670, *p* < 0.01) and with metabolic and liver-related markers, including VAI, ALT, and AST. MEDAS score showed inverse correlations with VAI and liver enzymes, whereas the cognitive restraint score was not significantly correlated with metabolic or liver-related parameters.

**Table 4:** Spearman correlations between behavioral eating patterns and metabolic-liver parameters.

Variable	1	2	3	4	5	6	7	8
MEQ	-	−0.647^**^	0.121	−0.707^**^	0.425^**^	−0.269^**^	−0.301^**^	−0.238^**^
TFEQ, EE	-	-	0.121	0.670^**^	−0.308^**^	0.196^**^	0.214^**^	0.158^*^
TFEQ, CR	-	-	-	−0.045	0.048	−0.046	−0.051	−0.044
TFEQ, UE	-	-	-	-	−0.356^**^	0.238^**^	0.223^**^	0.161^*^
MEDAS	-	-	-	-	-	−0.175^**^	−0.175^**^	−0.141^*^
VAI	-	-	-	-	-	-	0.728^**^	0.699^**^
ALT (U/L)	-	-	-	-	-	-	-	0.903^**^
AST (U/L)	-	-	-	-	-	-	-	-

Values are Spearman’s rho correlation coefficients (r). *p < 0.05, **p < 0.01. MEQ, Mindful Eating Questionnaire; MEDAS, Mediterranean Diet Adherence Screener; TFEQ, Three-Factor Eating Questionnaire; EE, emotional eating; CR, cognitive restraint; UE, uncontrolled eating; VAI, visceral adiposity index; ALT, alanine aminotransferase; AST, aspartate aminotransferase.

**Figure 1:**
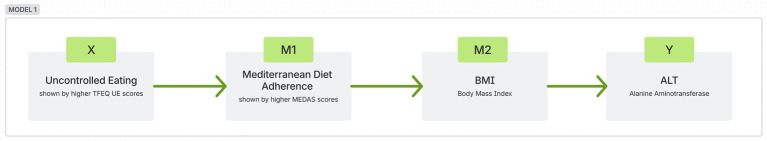
Main, interaction, and conditional direct and indirect effects of eating behaviors on serum ALT levels (Model 1).

As shown in Table 5 and Figure 1, serial mediation analyses evaluated the direct and indirect associations between uncontrolled eating scores and serum ALT levels, with MEDAS and BMI specified as sequential mediators and MAFLD status included as a moderator. In the overall sample, uncontrolled eating was negatively associated with MEDAS (B = −0.102, *p* < 0.001) and positively associated with BMI (B = 0.177, *p* = 0.003). BMI was positively associated with ALT levels (B = 3.705, *p* < 0.001), whereas the direct association between uncontrolled eating and ALT was not statistically significant (B = 0.541, *p* = 0.179).

**Table 5:** Main, interaction, and conditional direct and indirect effects of eating behaviors on serum ALT levels (Model 1).

Pathway	Overall		Group interaction		Non-MAFLD group		MAFLD group	
	B	*p*	B	*p*	B	*p*	B	*p*
X ➔ M1	**−0.102**	**<0.001**	−0.006	0.893	**−0.094**	**0.008**	**−0.100**	**<0.001**
X ➔ M2	**0.177**	**0.003**	−0.176	0.100	**0.212**	**0.011**	0.073	0.254
M1 ➔ M2	**−0.543**	**0.005**	−0.526	0.113	−0.085	0.730	**−0.649**	**0.002**
M1 ➔ Y	−0.808	0.526	−2.690	0.179	0.896	0.109	−1.375	0.388
M2 ➔ Y	**3.705**	**<0.001**	1.221	0.138	0.215	0.383	**1.638**	**0.008**
Direct path	0.541	0.179	0.570	0.385	0.190	0.324	0.675	0.152

Pathway	B	Lower	Upper	IMM	LLCI	ULCI	B	Lower	Upper	B	Lower	Upper
Indirect path 1	0.082	−0.177	0.356	0.258	−0.131	0.627	−0.084	−0.211	0.043	0.138	−0.232	0.483
Indirect path 2	**0.655**	**0.185**	**1.164**	0.006	−0.256	0.309	0.046	−0.077	0.156	0.119	−0.077	0.423
Indirect path 3	**0.205**	**0.065**	**0.383**	**0.090**	**0.003**	**0.233**	0.002	−0.014	0.024	**0.106**	**0.019**	**0.251**

This analysis examined the direct and indirect associations between uncontrolled eating behavior (X; TFEQ-UE scores) and serum alanine aminotransferase (ALT) levels (Y). Mediterranean diet adherence (M1; MEDAS score) and body mass index (M2; BMI) were specified as sequential mediators, forming a serial mediation pathway (X → M1 → M2 → Y). MAFLD status (non-MAFLD vs. MAFLD) was included as a moderator (W) to evaluate whether path coefficients differed by disease status. Overall (average) effects and group-specific conditional effects (non-MAFLD and MAFLD) were estimated using PROCESS Model 6. Interaction terms were derived from PROCESS Model 92 and are reported solely to assess whether the associations differed according to MAFLD status. Regression coefficients (B) and corresponding p values are reported for all direct paths. Indirect paths were defined as follows: Ind1 = TFEQ-UE → MEDAS → ALT; Ind2 = TFEQ-UE → BMI → ALT; Ind3 = TFEQ-UE → MEDAS → BMI → ALT. As *p* values are not provided for indirect effects, these effects are presented with 95% bias-corrected bootstrap confidence intervals (CI) based on 5,000 resamples. Interaction effects are evaluated using the index of moderated mediation (IMM) with 95% bootstrap confidence intervals. LLCI denotes the lower limit and ULCI the upper limit of the confidence interval. Age and sex were included as covariates in all regression equations. A statistically significant IMM indicates that the serial indirect effect differs by MAFLD status. Bold values indicate statistically significant results (*p* < 0.05).

In group-specific analyses, the association between BMI and ALT remained statistically significant in the MAFLD group (B = 1.638, *p* = 0.008) but not in the non-MAFLD group. The serial indirect effect through MEDAS and BMI differed between groups, as indicated by a statistically significant index of moderated mediation (IMM = 0.090; 95% bootstrap CI: 0.003–0.233). The indirect pathways through MEDAS alone and BMI alone were observed without a significant group interaction.

**Figure 2:**
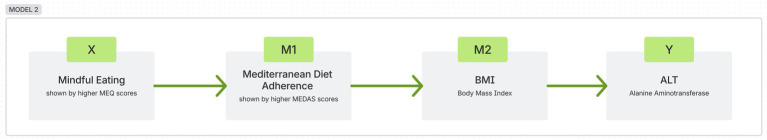
Main, interaction, and conditional direct and indirect effects of eating behaviors on serum ALT levels (Model 2).

As shown in Table 6 and Figure 2, serial mediation analyses examined the direct and indirect associations between MEQ scores and serum ALT levels, with MEDAS and BMI specified as sequential mediators and MAFLD status included as a moderator. In the overall sample, MEQ was positively associated with MEDAS (B = 0.054, *p* < 0.001) and inversely associated with BMI (B = −0.115, *p* < 0.001). BMI was positively associated with ALT levels (B = 3.583, *p* < 0.001), whereas the direct association between mindful eating scores and ALT did not reach statistical significance (B = −0.344, *p* = 0.059). In group-specific analyses, the association between BMI and ALT remained statistically significant in the MAFLD group (B = 1.614, *p* = 0.010) but not in the non-MAFLD group. The indirect effects through BMI alone and through the serial pathway involving MEDAS and BMI were statistically significant in the MAFLD group but not in the non-MAFLD group. However, the indices of moderated mediation were not statistically significant.

**Table 6:** Main, interaction, and conditional direct and indirect effects of eating behaviors on serum ALT levels (Model 2).

Pathway	Overall		Group interaction		non-MAFLD group		MAFLD group	
	B	*p*	B	*p*	B	*p*	B	*p*
X ➔ M1	**0.054**	**<0.001**	0.009	0.587	**0.047**	**0.002**	**0.056**	**<0.001**
X ➔ M2	**−0.115**	**<0.001**	0.030	0.527	**−0.071**	**0.049**	**−0.059**	**0.045**
M1 ➔ M2	**−0.385**	**0.046**	−0.422	0.216	−0.099	0.695	**−0.546**	**0.012**
M1 ➔ Y	−0.434	0.738	−2.816	0.171	1.037	0.066	−1.420	0.388
M2 ➔ Y	**3.583**	**<0.001**	1.208	0.141	0.199	0.409	**1.614**	**0.010**
Direct path	−0.344	0.059	−0.168	0.557	−0.132	0.105	−0.229	0.299

Pathway	B	Lower	Upper	IMM	LLCI	ULCI	B	Lower	Upper	B	Lower	Upper
Indirect path 1	−0.024	−0.168	0.122	−0.143	−0.363	0.074	0.049	−0.010	0.131	−0.080	−0.279	0.126
Indirect path 2	**−0.412**	**−0.633**	**−0.207**	−0.060	−0.218	0.049	−0.014	−0.055	0.026	**−0.095**	**−0.256**	**−0.004**
Indirect path 3	−0.075	−0.154	0.000	−0.040	−0.106	0.003	−0.001	−0.012	0.007	**−0.050**	**−0.119**	**−0.007**

This analysis examined the direct and indirect associations between mindful eating (X; assessed by MEQ scores) and serum alanine aminotransferase (ALT) levels (Y). Mediterranean diet adherence (M1; MEDAS score) and body mass index (M2; BMI) were specified as sequential mediators, forming a serial mediation pathway (X → M1 → M2 → Y). MAFLD status (non-MAFLD vs. MAFLD) was included as a moderator (W) to evaluate whether path coefficients differed according to disease status. Overall (average) effects and group-specific conditional effects (non-MAFLD and MAFLD) were estimated using PROCESS Model 6. Interaction terms were derived from PROCESS Model 92 and are reported solely to assess whether the associations differed by MAFLD status. Regression coefficients (B) and corresponding *p* values are reported for all direct paths. Indirect paths were defined as follows: Ind1 = Mindful eating → MEDAS → ALT; Ind2 = Mindful eating → BMI → ALT; Ind3 = Mindful eating → MEDAS → BMI → ALT. As p values are not provided for indirect effects, these effects are presented with 95% bias-corrected bootstrap confidence intervals (CI) based on 5,000 resamples. Interaction effects are evaluated using the index of moderated mediation (IMM) with 95% bootstrap confidence intervals. LLCI denotes the lower limit and ULCI the upper limit of the confidence interval. Age and sex were included as covariates in all regression equations. A statistically significant IMM indicates that the serial indirect effect differs by MAFLD status. Bold values indicate statistically significant results (*p* < 0.05).

## 4 Discussion

In this study, participants with MAFLD presented a more adverse metabolic and behavioral profile than non-MAFLD individuals, including higher BMI, waist circumference, VAI, and liver enzyme levels, together with lower mindful eating and Mediterranean diet adherence and higher uncontrolled and emotional eating. Correlation analysis showed mindful eating was inversely related to adiposity and liver enzymes, while uncontrolled and emotional eating were positively associated. Serial mediation analyses indicated that the indirect effects of eating behaviors on ALT through Mediterranean diet adherence and BMI were more apparent in the MAFLD group than in healthy controls. Considering the evolving terminology in fatty liver disease research, the findings of the present study are interpreted within the MAFLD framework, despite most of the existing literature using the NAFLD definition.

In this study, MAFLD was accompanied by a more unfavorable adiposity-related profile, encompassing increased BMI, central obesity, and higher VAI values, compared with healthy individuals. Consistent with this pattern, multivariable regression analyses indicated significant associations of both BMI and VAI with MAFLD status in separate models, in line with previous evidence (39–41). VAI has been proposed as an indicator of metabolic dysfunction, and its association with MAFLD has gained increasing attention in recent years. Current literature suggests that VAI may serve as a significant marker of MAFLD risk. In a four-year prospective cohort study involving 4,809 individuals, Xu et al. (2018) reported that individuals in the highest VAI quartile had a 2.13-fold higher risk of developing MAFLD compared with those in the lowest quartile (37). Similarly, Okamura et al. (2020) found a 3.69-fold higher risk in men and a 4.93-fold higher risk in women in the highest VAI quartile in a Japanese cohort (42). Consistent findings were also reported in an NHANES-based analysis by Li et al. (2022), demonstrating a significant positive association between VAI and MAFLD (43).

Individuals with MAFLD demonstrated less favorable eating behavior profiles, characterized by lower mindful eating and higher uncontrolled and emotional eating scores. Although differences in age distribution between groups should be considered when interpreting eating behavior scores, these differences were no longer statistically significant after controlling for age, sex, and BMI, and eating behavior scores did not remain independently associated with MAFLD after the inclusion of adiposity measures (VAI and BMI) in logistic regression models. Nevertheless, considering the tendency for chronic metabolic diseases to emerge with increasing age, it should be taken into account that the case and control groups in the present study were not individually age matched. Notably, the persistence of adiposity- and liver enzyme-related differences after age adjustment suggests that these associations may be largely independent of age effects. In this context, emotional eating has been proposed as an important behavioral risk factor in the development of MAFLD, with its association partly explained by obesity (44). Sugiyama et al. compared obese and non-obese individuals with MAFLD to healthy controls using sex-stratified analyses and reported significantly higher emotional eating scores only among obese women with MAFLD, whereas no significant differences were observed in non-obese women or men compared with controls (45).

Another key behavioral risk factor that may contribute to the development of MAFLD is uncontrolled eating. Individuals with MAFLD are more likely to exhibit uncontrolled eating patterns compared to healthy controls (46). Rapid and uncontrolled eating may lead to a weaker anorexigenic gut hormone response that fails to effectively trigger satiety signals, thereby promoting binge-eating behavior (47). In a pilot study by Zhang et al., individuals diagnosed with MAFLD showed a significantly higher tendency toward binge eating compared with the general population (23.1% vs. 2.6%) (48). Another study in individuals with MAFLD found that higher levels of binge eating were associated with higher BMI (49).

Mindful eating is conceptualized as an approach that enhances individuals’ awareness of emotional or uncontrolled eating behaviors triggered by stress, mood states, or external cues, thereby facilitating the transformation of automatic responses into more conscious and healthier food choices (50). In the present study, mindful eating scores were significantly and negatively associated with emotional and uncontrolled eating in both healthy individuals and those with MAFLD. These findings are partially consistent with previous research reporting inverse relationships between mindful eating and maladaptive eating behaviors among adults (51). In the multivariate regression analysis conducted using structural equation modeling, self-regulation in eating behaviors among university students was found to be positively associated with mindfulness and negatively associated with uncontrolled and emotional eating. Interestingly, the “awareness” subscale score of the MEQ was positively associated with emotional eating. While this finding may initially appear contradictory to our study, it is important to note that the “awareness” subscale in that study reflects sensitivity to emotional cues during eating episodes. Considering the sample consisted of university students, it is plausible that although these individuals are aware of their internal emotional states, they may lack the necessary coping mechanisms. This could potentially lead to paradoxical effects on emotional and uncontrolled eating behaviors (52). In line with this notion, individuals with MAFLD have been reported to exhibit uncontrolled eating behaviors despite being aware of recommended dietary practices, pointing to a potential gap between knowledge and behavior adoption (46).

In this study, uncontrolled and mindful eating behaviors showed opposite associations with adherence to the Mediterranean diet across both groups, with uncontrolled eating being negatively associated and mindful eating positively associated with diet adherence. This finding is consistent with previous evidence indicating that emotional, uncontrolled, and mindless eating are linked to higher consumption of energy-dense, nutrient-poor foods and may act as barriers to adherence to the Mediterranean diet (53). Our results are in line with a study conducted in Türkiye by Görgülü Doğan and Tengilimoğlu-Metin (2023), which reported a positive association between the “eating discipline” subscale of the MEQ and MEDAS scores, as well as increased consumption of green vegetables, legumes, and dairy products among individuals with higher mindful eating levels (54). Similarly, findings from the large-scale NutriNet-Santé cohort study indicated that higher mindful eating scores were positively associated with greater adherence to the Mediterranean diet, lower consumption of ultra-processed foods, and higher preference for organic products (55). In another study by Christodoulou et al., a significant positive correlation was observed between mindful eating scores and adherence to the Mediterranean lifestyle, with noted benefits for body weight management (56).

When considered collectively, these findings suggest that mindful eating may represent a behavioral characteristic associated with greater adherence to healthier dietary patterns. In this context, mindful eating may provide a framework that supports individuals in more consciously evaluating their eating behaviors during periods of impaired eating control. Within the existing literature, mindfulness-based approaches have been reported to be associated with the regulation of problematic eating behaviors, including emotional and binge eating. These associations have been discussed in relation to increased sensitivity to internal hunger and satiety cues, as well as alterations in functional connectivity between the hypothalamus, reward-related brain regions, and the default mode network (DMN) (57–59).

Nevertheless, neither eating behavior scores nor adherence to the Mediterranean diet demonstrated a direct association with serum ALT levels in either group. In serial mediation analyses, adherence to the Mediterranean diet was inversely associated with BMI, while BMI was positively associated with ALT levels; notably, these pathways reached statistical significance only in the MAFLD group. A meta-analysis of cohort studies has shown that obesity is an independent risk factor for the development of MAFLD, with obese individuals exhibiting an approximately 3.5-fold higher risk compared with those of normal weight (60). In addition, a study describing a U-shaped association between BMI and liver enzymes reported that stratified analyses indicated a modifying effect of MAFLD on the relationship between BMI and AST and ALT levels, with more pronounced associations observed at higher BMI ranges among individuals with MAFLD (61). However, another study suggested that this association may be sex-specific, being evident in men but not in women (62). Taken together, the findings of the present study suggest that interpreting MAFLD within the framework of metabolically unhealthy obesity may be appropriate, and that the relationships between eating behaviors and liver health may be shaped through adiposity-related pathways (63).

Consistent with these observations, indirect associations between eating behaviors and ALT levels were identified only through pathways involving BMI. Indirect effects mediated solely by MEDAS were not statistically significant, whereas BMI-mediated pathways and the sequential MEDAS → BMI indirect pathway reached statistical significance only in the MAFLD group. This pattern suggests that the association between adherence to the Mediterranean diet and liver health may be linked primarily to adiposity-related pathways rather than to direct pathways. Indeed, although unadjusted analyses showed lower adherence to the Mediterranean diet among individuals with MAFLD, MEDAS score was not independently associated with MAFLD status after adjustment for age, sex, and BMI, nor in multivariable logistic regression models incorporating adiposity and eating behavior variables.

In line with these results, the Swiss CoLaus cohort study prospectively examined the association between adherence to the Mediterranean diet and incident hepatic steatosis among adults free from clinically manifest steatosis at baseline. Over a mean follow-up of 5.3 years, an inverse association was observed between Mediterranean diet score and the risk of hepatic steatosis defined by Fatty Liver Index (FLI ≥ 60). However, this association was attenuated to the null after adjustment for general and central adiposity assessed by BMI and waist circumference. In contrast, no significant association was found when hepatic steatosis was defined using the NAFLD score (64). Similarly, cross-sectional analyses from two population-based adult cohorts, Fenland (UK) and CoLaus (Switzerland), reported that higher adherence to a pyramid-based Mediterranean diet score was associated with a lower prevalence of hepatic steatosis assessed by ultrasonography and FLI. Nevertheless, these associations were consistently weakened after adjustment for BMI, suggesting that the inverse relationship between Mediterranean diet adherence and hepatic steatosis is largely explained by adiposity (65). Moreover, a cross-sectional study conducted among adults with MAFLD reported no significant association between adherence to the Mediterranean diet and hepatic steatosis or fibrosis severity (66).

In contrast, previous studies have reported stronger associations. Barrea et al. demonstrated lower adherence to the Mediterranean diet in individuals with MAFLD and identified the PREDIMED score as the strongest predictor of the Fatty Liver Index (FLI), with a score below six indicating an increased risk of NAFLD (13). Similarly, in the Amol Cohort Study, an inverse association was observed between adherence to the Mediterranean diet and NAFLD, and this association was evident in individuals both with and without abdominal obesity (67). In the Ravansar Noncommunicable Disease cohort, individuals in the highest tertile of Mediterranean diet adherence exhibited lower FLI values, and the probability of hepatic fibrosis decreased significantly across increasing tertiles of adherence (68). In a retrospective analysis, after adjustment for potential confounding variables, higher adherence to the Mediterranean diet was found to be significantly associated with lower odds of MASLD (69). Evidence from cross-sectional studies and meta-analyses supports that higher adherence to the Mediterranean diet is associated with favorable changes in liver enzymes, cardiometabolic indicators (including the VAI and FLI), and overall metabolic health (13, 70). Moreover, in the context of MAFLD, the protective role of the Mediterranean diet has been more clearly demonstrated in longitudinal and interventional studies, which have reported reductions in hepatic steatosis severity and marked decreases in liver fat content (15, 68, 69, 71). However, as highlighted in the systematic review and meta-analysis by Haigh et al., much of the existing evidence on the effectiveness of the Mediterranean diet derives from studies conducted in Mediterranean regions with favorable food environments and culturally embedded dietary practices. Consequently, the observed effects on disease outcomes may be influenced by baseline adherence to the Mediterranean diet, potentially limiting the generalizability of these findings to populations with different habitual dietary patterns (70).

The limited mediating role of Mediterranean diet adherence observed in the present study may be related to its cross-sectional design, which inherently restricts the ability to establish temporal ordering and causal mechanisms, particularly in mediation analyses. In addition, adherence to the Mediterranean diet was assessed using a screening-based, self-reported instrument (MEDAS) rather than objective dietary intake measures, which further limits the interpretation of the Mediterranean diet score as a true mediating variable. Indeed, in a study in which diet quality was assessed using more detailed methods such as food frequency questionnaires, and individuals with and without MAFLD were compared, more pronounced associations between diet quality and BMI were reported (72). This methodological difference may partly explain the limited mediating effects observed in the present study, as MEDAS may not adequately capture absolute intake amounts that are more directly related to adiposity.

Notably, in the model with uncontrolled eating as the predictor, the serial indirect path through Mediterranean diet adherence and BMI differed significantly by MAFLD status, as indicated by a significant index of moderated mediation. This result suggests that the indirect association between uncontrolled eating and ALT levels via diet adherence and adiposity was more pronounced in individuals with MAFLD than in those without MAFLD. In contrast, mediation models with mindful eating as the predictor did not show significant moderated mediation, despite the presence of significant BMI-mediated indirect paths in the MAFLD group. The finding that indirect effects were observed exclusively in the MAFLD group may be explained by the greater influence of adiposity and metabolic dysregulation on the relationship between uncontrolled eating behaviors and liver enzymes in this population. Increased metabolic vulnerability, hepatic steatosis, and hepatic sensitivity in MAFLD may have contributed to the stronger emergence of BMI-mediated pathways. In contrast, greater metabolic flexibility and compensatory mechanisms in individuals without MAFLD may have attenuated these associations (45, 73). Nevertheless, unmeasured factors such as physical activity level, socioeconomic characteristics, metabolic comorbidities, and medication use may influence both eating behaviors and liver biomarkers and could therefore contribute to residual confounding, which should be considered when interpreting these findings.

This study has several limitations. First, its cross-sectional design precludes establishing causality between eating behaviors, adiposity, and liver health; longitudinal studies are needed to clarify the direction and temporal dynamics of these associations. In this regard, although serial mediation analyses were applied, the observed indirect effects should be interpreted as statistical associations rather than evidence of temporal ordering or underlying mechanistic pathways. In addition, the potential influence of unmeasured confounders such as physical activity levels, socioeconomic factors, metabolic comorbidities, and medication use cannot be ruled out and may have affected both eating behaviors and liver-related biomarkers. Furthermore, dietary intake and eating behaviors were assessed through self-reported questionnaires, which may be prone to recall bias and social desirability effects. The absence of objective dietary intake measures also limits the interpretation of Mediterranean diet adherence as a definitive mediator within the proposed analytical framework. Moreover, information regarding the severity or stage of MAFLD was not available, as diagnostic procedures and disease staging were not performed within the scope of the study. Different degrees of hepatic steatosis or fibrosis may differentially influence eating behaviors, adiposity, and liver biomarkers; therefore, the observed associations may vary across disease stages. Although the sample size was adequate and statistically well powered for the primary analyses, the inability to perform subgroup analyses according to MAFLD severity limited subgroup comparisons and reduced the generalizability of the findings across different disease stages and other populations.

## 5 Conclusion

This study provides new insights into the complex interplay between eating behaviors, dietary adherence, obesity, and liver health. Our findings indicate that the associations between uncontrolled and mindful eating behaviors and serum ALT levels are not direct; rather, these relationships are largely mediated by adherence to the Mediterranean diet and BMI, with these pathways being more pronounced among individuals with MAFLD and in models where uncontrolled eating was specified as the predictor. These results suggest that supporting mindful eating behaviors and improving adherence to the Mediterranean diet may contribute to reductions in adiposity and the promotion of liver health, particularly in metabolically vulnerable populations.

Future research should employ longitudinal and interventional study designs incorporating objective measures of liver fat to more clearly elucidate causal directions and to evaluate the effectiveness of mindfulness-based approaches and diet quality–focused strategies in the prevention and management of MAFLD. In addition, the inclusion of larger and more diverse populations would further enhance the clinical and public health relevance of these findings.

## Data Availability

The raw data supporting the conclusions of this article will be made available by the authors, without undue reservation.
